# Differential expression of alternatively spliced transcripts related to energy metabolism in colorectal cancer

**DOI:** 10.1186/s12864-016-3351-5

**Published:** 2016-12-28

**Authors:** Anastasiya Vladimirovna Snezhkina, George Sergeevich Krasnov, Andrew Rostislavovich Zaretsky, Alex Zhavoronkov, Kirill Mikhailovich Nyushko, Alexey Alexandrovich Moskalev, Irina Yurievna Karpova, Anastasiya Isaevna Afremova, Anastasiya Valerievna Lipatova, Dmitriy Vladimitovich Kochetkov, Maria Sergeena Fedorova, Nadezhda Nikolaevna Volchenko, Asiya Fayazovna Sadritdinova, Nataliya Vladimirovna Melnikova, Dmitry Vladimirovich Sidorov, Anatoly Yurievich Popov, Dmitry Valerievich Kalinin, Andrey Dmitrievich Kaprin, Boris Yakovlevich Alekseev, Alexey Alexandrovich Dmitriev, Anna Viktorovna Kudryavtseva

**Affiliations:** 1 Engelhardt Institute of Molecular Biology, Russian Academy of Sciences, Moscow, Russia; 2 National Medical Research Radiological Center, Ministry of Health of the Russian Federation, Moscow, Russia; 3 Shemyakin-Ovchinnikov Institute of Bioorganic Chemistry, Russian Academy of Sciences, Moscow, Russia; 4 Insilico Medicine, Inc., Emerging Technology Centers, Johns Hopkins University Eastern Campus, Baltimore, Maryland USA; 5 Moscow Institute of Physics and Technology, Dolgoprudny, Russia; 6 State Hospital №57, Moscow, Russia; 7 A.V. Vishnevsky Institute of Surgery, Moscow, Russia

**Keywords:** Alternative splicing, Energy metabolism, Tumor-specific alternative mRNA transcripts, Colorectal cancer, Adenocarcinoma

## Abstract

**Background:**

Colorectal cancer (CRC) is one of the most common malignant tumors worldwide. CRC molecular pathogenesis is heterogeneous and may be followed by mutations in oncogenes and tumor suppressor genes, chromosomal and microsatellite instability, alternative splicing alterations, hypermethylation of CpG islands, oxidative stress, impairment of different signaling pathways and energy metabolism. In the present work, we have studied the alterations of alternative splicing patterns of genes related to energy metabolism in CRC.

**Results:**

Using CrossHub software, we analyzed The Cancer Genome Atlas (TCGA) RNA-Seq datasets derived from colon tumor and matched normal tissues. The expression of 1014 alternative mRNA isoforms involved in cell energy metabolism was examined. We found 7 genes with differentially expressed alternative transcripts whereas overall expression of these genes was not significantly altered in CRC. A set of 8 differentially expressed transcripts of interest has been validated by qPCR. These eight isoforms encoded by *OGDH*, *COL6A3*, *ICAM1*, *PHPT1*, *PPP2R5D*, *SLC29A1*, and *TRIB3* genes were up-regulated in colorectal tumors, and this is in concordance with the bioinformatics data. The alternative transcript NM_057167 of *COL6A3* was also strongly up-regulated in breast, lung, prostate, and kidney tumors. Alternative transcript of *SLC29A1* (NM_001078177) was up-regulated only in CRC samples, but not in the other tested tumor types.

**Conclusions:**

We identified tumor-specific expression of alternative spliced transcripts of seven genes involved in energy metabolism in CRC. Our results bring new knowledge on alternative splicing in colorectal cancer and suggest a set of mRNA isoforms that could be used for cancer diagnosis and development of treatment methods.

**Electronic supplementary material:**

The online version of this article (doi:10.1186/s12864-016-3351-5) contains supplementary material, which is available to authorized users.

## Background

Colorectal cancer (CRC) is the third most common cancer in the world [1]. It accounts for more than 1,360,000 new cancer incidences and about 9% of all cancer deaths worldwide [2], [http://gco.iarc.fr/]. The risk of CRC increases with age, and CRC incidence rates are higher among males, than females [3]. CRC starts in either the colon or the rectum and is represented by several histological types. More than 90% of CRC are adenocarcinomas [4]. Generally CRC patients are characterized by a lack of clinical symptoms in early stages, and this leads to poor prognosis and high mortality rate [5]. Approximately 20% of patients with CRC have already developed metastatic disease at the time of diagnosis [6, 7]. Colorectal cancer metastases are found in the liver, lung, skin, and brain [8]. The median survival of patients with advanced metastatic disease is less than 24 months [9]. Although the medical management of CRC has improved, there are limited therapeutic options for advanced CRC. Identification of new therapeutic targets and biomarkers is imperative for the development of CRC therapies and diagnosis.

Energy metabolism in cancer cells is characterized by increased glucose uptake and aerobic glycolysis [10]. Even in the presence of oxygen, most cancer cells produce lactate instead of oxidation of glycolytic pyruvate in the mitochondrial tricarboxylic acid (TCA) cycle [11, 12]. This phenomenon was originally observed by Otto Warburg and termed the “Warburg effect” [13]. Increased aerobic glycolysis is associated with the alteration of gene expression, protein modifications and mutations [14]. Warburg effect is the tumor adaptation mechanism to oxidative stress and hypoxia [15]. Each type of cancer is characterized by a distinct metabolic signature due to its unique transformation process [16, 17]. Elucidation of the metabolic specificities of several cancers and metabolic differences between cancer and normal cells has provided important biomarker findings [18, 19].

The aim of the study was to identify tumor-associated expression of alternatively spliced transcripts related to energy metabolism in CRC. Obtained data suggest several ones that could be involved in the development of CRC through altered energy metabolism. Identified tumor-specific mRNA isoforms may be used for the development of cancer diagnosis and treatment methods.

## Methods

### Bioinformatics analysis

We analyzed TCGA RNA-Seq datasets (read counts) for colon cancer using CrossHub software [20]. Here is a brief description of TCGA RNA-Seq Version 2 pipeline. Illumina reads were aligned to hg19 UCSC reference genome using MapSplice [21]. The alignment results were translated to transcriptome coordinates prior to transcript level quantification using the UNC Bioinformatics Utilities (https://github.com/mozack/ubu). RSEM was used to estimate gene and transcript abundances [22]. The further analysis of read count data was performed using CrossHub [20]. Read counts were normalized with TMM (trimmed mean of M-values) method and then two expression test were performed: for two pools of samples and for paired samples only (paired samples comprise only about 10% of all TCGA samples). We excluded lowly expressed isoforms; only genes with at least 70 reads in each of 50% samples (either normal or tumor) have passed expression level threshold. Then comparison of trimmed (4% from each tail) mean of expression values between normal and tumor tissues using *t*-test was performed. Behjamini-Hochberg *p*-value adjustment was performed in order to calculate FDR. We selected only isoforms with concordant results in paired and pooled tests.

The selection of genes participating cell energy metabolism was performed using Gene Ontology database and the following keywords: glucose, glycolytic, glycolysis, cell respiration, respiratory, TCA cycle, oxidative phosphorylation, Krebs. Finally, a set of 277 genes with 1014 alternatively spliced transcripts was selected for the further analysis. Unfortunately, TCGA read count data were derived for the previous genome assembly (hg19) using UCSC genome annotation. When possible, UCSC transcript identifiers were converted to RefSeq accession numbers. Using CrossHub, we analyzed associations with disease stage, follow-up status, TNM indexes. We paid special attention to the alternatively spliced transcripts that are strongly overexpressed in colon tumors against the background of low changes in overall gene expression level or its down-regulation.

### Tissue specimens

A total of 40 colorectal, 30 breast, 30 non-small cell lung, 30 prostate, and 30 kidney cancer specimens and matched morphologically normal tissues were obtained after surgical resection prior to radiation or chemotherapy. The samples were frozen and stored in liquid nitrogen. The morphological classification of the tumor was performed according to the American Joint Committee on Cancer (AJCC) staging system [23]. Only samples with 70% or more tumor cells were studied. Written informed consent was obtained from all patients. The study was approved by The Ethics committee of Herzen Moscow Cancer Research Institute, Ministry of Health of the Russian Federation. The study was done in accordance with the principles outlined in the Declaration of Helsinki (1964). The sample information for colorectal tumors is presented in Table 1.

**Table 1:** Clinicopathologic characteristics of CRC patients

Characteristic	Total, n
Gender	
Male Female	23 17
Age	
≤ 60 > 60	9 31
Clinical stage	
I II III IV	3 13 4 20

### Isolation of RNA and cDNA synthesis

Total RNA was isolated from frozen tissues using RNeasy Mini kit (Qiagen, Germany) according to manufacturer's instructions. RNA quality was measured using the RIN method (RNA Integrity Number) on Agilent RNA Bioanalyzer 2100 (Agilent Technologies, USA). The RNA quantification was carried out on a NanoDrop 1000 (NanoDrop Technologies Inc., USA). cDNA synthesis was done using M-MLV Reverse Transcriptase (Thermo Fisher Scientific, USA) and random hexamers.

### Quantitative PCR

Gene and transcript expression levels were estimated by quantitative PCR (qPCR) analysis. All probes contained the dye FAM at 5′-end and RTQ1 at 3′-end. Specific primer pairs and probes were synthesized for the alternative splicing isoforms of target genes (Table 2). Primers and probes for reference genes were designed as previously described [24, 25]. PCR was carried out in triplicates on AB 7500 Real-Time PCR System (Thermo Fisher Scientific, USA) following the manufacturer's instructions. PCR program was as follows: 10 min at 95 °C and then 50 two-step cycles 15 s at 95 °C and 60 s at 60 °C. The total reaction volume was 20 μL in triplicate. PCR products were analyzed in 2% agarose gels, purified and submitted for Sanger sequencing on ABI Prism 3100 Genetic Analyzer (Thermo Fisher Scientific, USA).

**Table 2:** Primer and probe sets for qPCR analysis

Gene	Transcripts (UCSC database)	Direction	Primer sequence, 5’ → 3’	Amplicon size, bp
*COL6A3*	universal	Forward Reverse Probe	TCCAAGCCAAGAACGCAGA TGACGCCCTCAGAGCCAT ACGGAGCACCAGCACCAGTTTCAGG	204
	uc002vwo / NM_057167 uc002vwq / NM_057165 (not expressed according to RNA-Seq data)	Forward Reverse Probe	ACACACGCCTTCAGGTTTGC GACTGCGAAATTGACACTTCCG CAGCAGCAGCAAGCAGCACAAGACTC	218
*ICAM1*	universal	Forward Reverse Probe	CACCCCAGAGGACAACGG TGGCACATTGGAGTCTGCTG CCGGCCAGCTTATACACAAGAACCAGA	180
	uc010xle / AK301412	Forward Reverse Probe	CGCTATGGCTCCCAGCAG TGGCAGCGTAGGGTAAGGTTC TCTGTTCCCAGGGACTCCAGAACGG	147
*OGDH*	universal	Forward Reverse Probe	AAGTCTAGTGAGAATGGCGTGGACT CAAGGTAATGTTCCTGTCGGTGAC TTCAGCCGCCCTCTGTGTGGCAT	219
	uc011kby / AK296400	Forward Reverse Probe	GATGTACTGTGCTTGGCTGGAAA GATGATCTCCCGCAGAGGAAGT CAGGCCATAGAACCCTTATGTACACTTTTGGG	147
*PHPT1*	universal	Forward Reverse Probe	AAGTACCCCGACTACGAGGTCA GGCTCTGAAGTGGCTGCTG CTAACGACGGCTACTGAGCACTCCCA	92
	uc004cjq / NM_014172	Forward Reverse Probe	AAGGCTGCGACTGTGAGTGTCT CTCAGTTGAAATGGCGTGCTG CGGCTATTCCATGGCCTATGGTCCTG	122
*PPP2R5D*	universal	Forward Reverse Probe	CGGGACTTCCTCAAGACCATT ATGATGCTGCCCAGGATCTC CACATCTTCTACAGGTTCATCTACGAGACGGA	161
	uc010jyd / NM_180977	Forward Reverse Probe	GGCCGAGATGCCCTATAAACT TTGAGTCCTGCCCGCTTC CTTCTGGATAAACAGCTCCTTCTCCTTTTTCAG	138
*SLC29A1*	universal	Forward Reverse Probe	CATTTTGACCATCATCTGTTACCTG GGTCCAACTTGGTCTCCTGCT CCCCGCCTGGAATTCTACCGCTACTA	107
	uc003owz / NM_001078177	Forward Reverse Probe	GAGCCTGAGGACCCTGCG CGATGGGGATCACCCGTC CAACGTGACCGCAGCCTGTTTTAGGC	127
*TRIB3*	universal	Forward Reverse Probe	GCGTGATCTCAAGCTGTGTCG GCCTTGCCCGAGTATGAGG CAGCTTCTTCCTCTCACGGTCAGCGAAG	183
	uc002wdm / NM_021158	Forward Reverse Probe	ACCTGCTGGTGCCCTGGAG CGTTTCTGGACGGGACGCT ACGGGGCGAGATGCGAGCCACC	168
	uc002wdn / AK297546	Forward Reverse Probe	GTCATCCCAGCCTCGAACCT TCCAACTCCAACCGCTTCTTC TACCTGGCAACAGATGCGAGCCACC	191

Only UCSC hg19 target transcripts are listed

### Analysis of qPCR data

The Relative Quantitation software (Thermo Fisher Scientific, USA) and ATG (Analysis of Transcription of Genes) tool were used to analyze the obtained qPCR data taking into account the efficiency of the PCR amplification [26, 27]. The expression levels of target genes were normalized to *GAPDH* and *ACTB* reference genes and finally relative (T/N) expression level of target genes was calculated using *ΔΔC*_*t*_ method. The relative inner variability between mRNA levels of reference genes do not exceed two times, and, therefore, 2-fold or more expression alterations of the target genes/isoforms were considered significant. Inter-group and intra-group comparisons were performed using nonparametric Wilcoxon/Mann-Whitney U–test and Kruskal-Wallis test. Differences with *p* < 0.05 were considered statistically significant. The statistical procedures were performed with BioStat software (AnalystSoft Inc., USA).

## Results

### Analysis of TCGA data with CrossHub

Differential expression profiles were derived for 277 genes (1014 alternatively spliced transcripts) which participate in cell energy metabolism. 285 tumor and 41 matched normal colon tissue samples were used in the analysis. Among differentially expressed transcripts, we paid a special attention to the following eight mRNA isoforms, which were overexpressed in colon tumors: *OGDH* (uc011kby/AK296400), *COL6A3* (uc002vwo/NM_057167), *ICAM1* (uc010xle/AK301412), *PHPT1* (uc004cjq/NM_014172), *PPP2R5D* (uc010jyd/NM_180977), *SLC29A1* (uc003owz/NM_001078177), *TRIB3* (uc002wdm/NM_021158 and uc002wdn/AK297546). Most of them are minor mRNA isoforms that are selectively up-regulated against the background of intact or under-expressed other isoforms and intact expression of entire gene (sum across all transcripts). The list of LogFC, *p*-values, FDR, CPM, RNA-Seq expression profiles and associations with clinical characteristics is provided in the Additional file 1. Differential expression of these transcripts was further validated with qPCR. Most of the selected isoforms have unique splice event which do not occur in the other isoforms (e.g. exon boundaries). This allows accurate detection and quantification of these transcripts with spliced reads spanning this unique exon junction.

*OGDH* (oxoglutarate dehydrogenase) mRNA isoform uc011kby lacks three exons and this unique feature allows quantification of this transcripts with RNA-Seq data. Compared to the reference isoform (UniProt ID Q02218-1), the protein encoded with uc011kby transcript lacks three regions: 75–123 a.a., 139–172 a.a., and 211–263 a.a. Full-length isoform of *OGDH* is normally regulated by Ca^2+^, adenine nucleotides, and NADH. Mutations in the second region (D154A for Q02218-1) results in 6-fold decrease of calcium sensitivity [28]. Transcript uc003owz is very minor isoform of *SLC29A1* mRNA (CPM = 0.3 and 2.1 for normal and tumor accordingly). The first exon of this transcript almost completely covers CpG island in the promotor region of *SLC29A1*. Frequent induction of this minor isoform suggests altered mechanisms of *SLC29A1* expression regulation in colon tumors.

Two other transcripts of interest, uc010xle and uc010jyd are minor isoforms of *ICAM1* (Intercellular Adhesion Molecule 1) and *PPP2R5D* (Protein Phosphatase 2 Regulatory Subunit B' Delta) genes, respectively. Compared to the major isoforms, they do not include some exons. As the result, protein encoded with *ICAM1* uc010xle has deletion at 22–244 a.a. (UniProt P05362-1) which spans two Ig-like C2-type domains. The signal peptide sequence is almost completely kept (1–28 a.a.). This isoform is strongly overexpressed in tumor (CPM is 0.6 and 5.2 for normal and tumor) whereas major isoform is overexpressed only 1.5-times (CPM = 36 and 53). Protein encoded with *PPP2R5D* transcript uc010jyd has deletion at 10–107 a.a. (UniProt Q14738-1).

### Up-regulation of eight alternative mRNA transcripts in CRC

**Fig 1:**
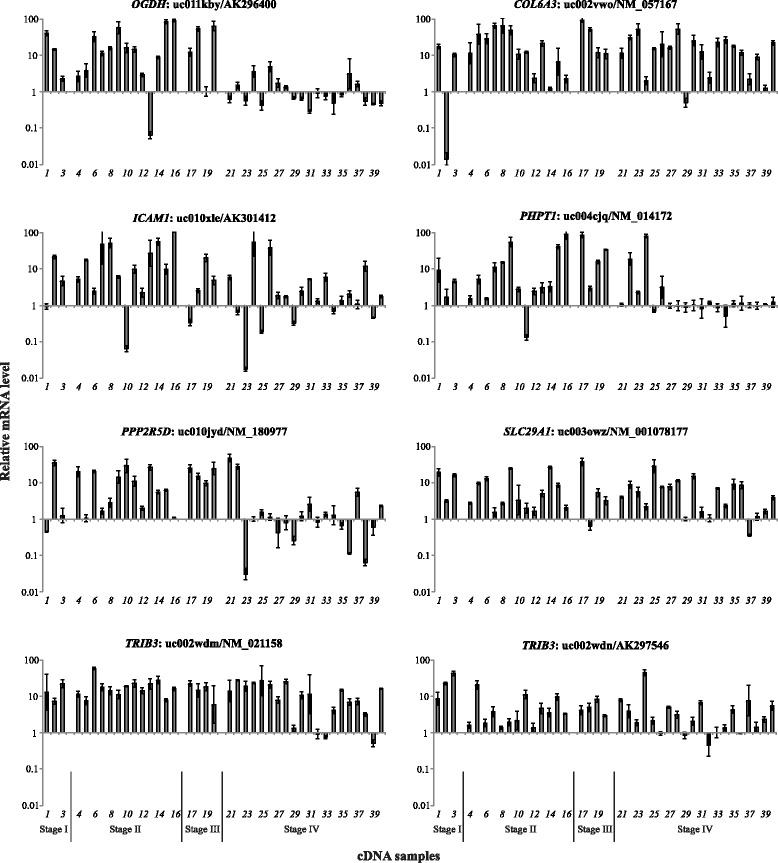
Up-regulation of eight alternative mRNA transcripts in CRC. *OGDH*: uc011kby/AK296400, *COL6A3*: uc002vwo/NM_057167, *ICAM1*: uc010xle/AK301412, *PHPT1*: uc004cjq/NM_014172, *PPP2R5D*: uc010jyd/NM_180977, *SLC29A1*: uc003owz/NM_001078177, *TRIB3*: uc002wdm/NM_021158 and uc002wdn/AK297546

Quantitative expression estimation was performed for eight alternative mRNA isoforms of seven genes involved in energy metabolism in CRC (Fig. 1). Bioinformatics results showed good concordance with qPCR results. All the transcripts showed up-regulation in more than 50% of CRC cases (Table 3). The highest frequency and extent of the mRNA level increase were observed for alternative transcripts of *COL6A3* (uc002vwo/ NM_057167, 90% and 10.6-fold average increase), *TRIB3* (uc002wdm/NM_021158, 90% and 10.5-fold average increase; uc002wdn/AK297546, 67.5% and 3.6-fold average increase), and *SLC29A1* (uc003owz/NM_001078177, 77.5% and 4.6-fold average increase) genes.

**Table 3:** Frequency of alterations and relative mRNA level of eight alternative mRNA transcripts in CRC

Genes	Frequency of mRNA level changes, %		Median of mRNA level changes, n-fold
	↑	↓	
*OGDH*			
uc011kby.1	52.5 (21/40)	15 (6/40)	3.1 ↑
*COL6A3*			
uc002vwo.2	90 (36/40)	5 (2/40)	10.6 ↑
*ICAM1*			
uc010xle.1	62.5 (25/40)	15 (6/40)	3.5 ↑
*PHPT1*			
uc004cjq.3	50 (20/40)	5 (2/40)	3.2 ↑
*PPP2R5D*			
uc010jyd.2	50 (20/40)	15 (6/40)	2.6 ↑
*SLC29A1*			
uc003owz.1	77.5 (31/40)	2.5 (1/40)	4.7 ↑
*TRIB3*			
uc002wdm.2 uc002wdn.2	90 (36/40) 67.5 (27/40)	2.5 (1/40) 2.5 (1/40)	10.5 ↑ 3.6 ↑

*Note:* qPCR data. ↓/↑: mRNA level decrease/increase. *P* < 0.05 for all cases

### Simultaneous up-regulation of alternative mRNA transcripts in colorectal, breast, lung, prostate, and kidney cancers

**Fig 2:**
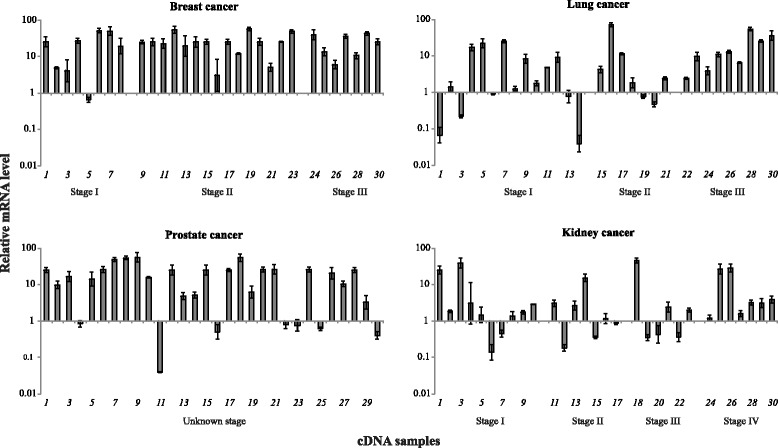
Up-regulation of the *COL6A3* alternative transcript uc002vwo/NM_057167 in breast, lung, prostate, and kidney cancers

To evaluate the possibility of tumor-specific expression of alternative transcripts, we analyzed their expression in breast, lung, prostate, and kidney cancers. We revealed the significantly increased expression of alternative mRNA isoform uc002vwo/NM_057167 of *COL6A3* in all these tumors (Fig. 2). The mRNA level of the transcript was up-regulated in 96.7% (29 of 30, *p* < 0.05) of breast, 63.3% (19 of 30, *p* < 0.05) of lung, 76.7% (23 of 30, *p* < 0.05) of prostate, and 50% (15 of 30, *p* < 0.05) of kidney cancer samples.

**Fig 3:**
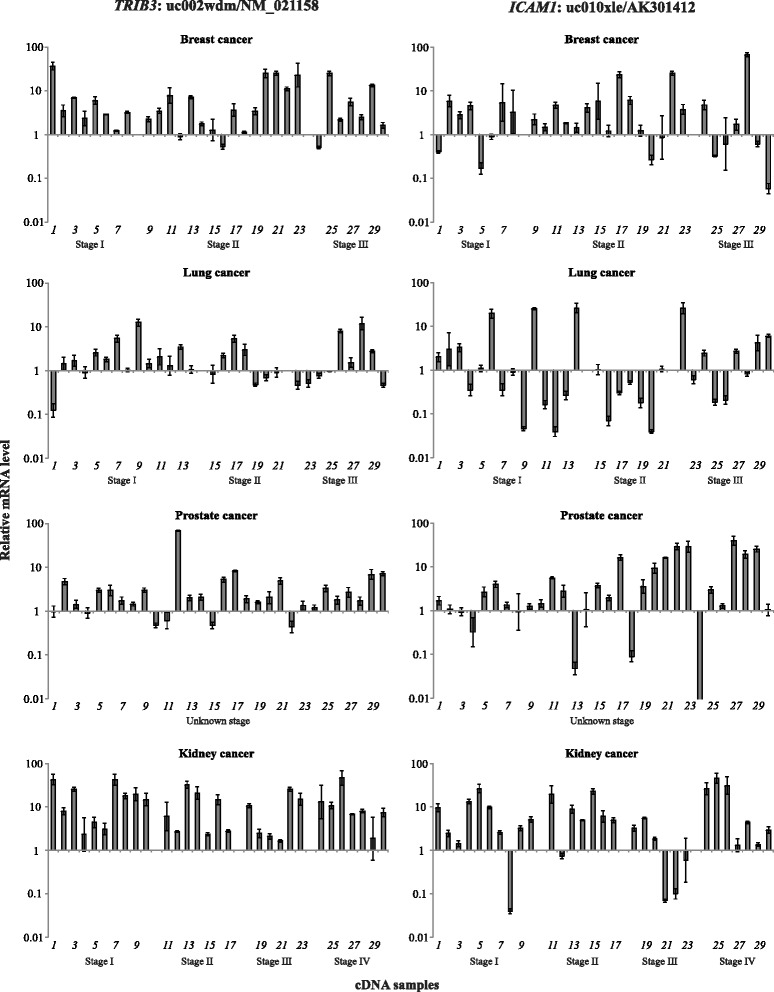
Relative mRNA levels of the *TRIB3* alternative transcript uc002wdm/NM_021158 and *ICAM1* alternative transcript uc010xle/AK301412 in breast, lung, prostate, and kidney cancers

**Fig 4:**
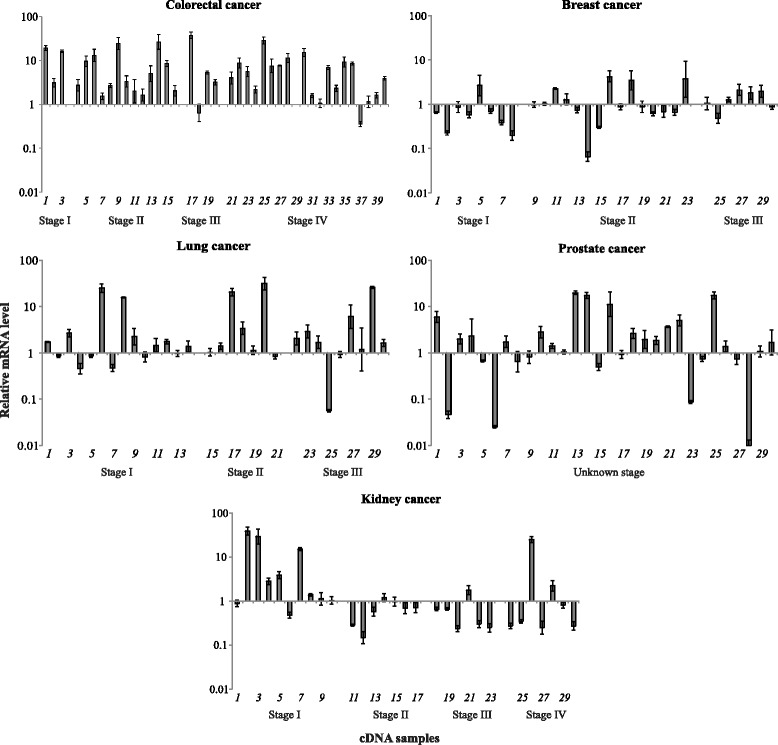
The relative mRNA level of the *SLC29A1* alternative transcript uc003owz/NM_001078177 in colorectal, breast, lung, prostate, and kidney cancers

We also observed the increased expression of two alternative transcripts (*TRIB3*: uc002wdm/NM_021158 and *ICAM1*: uc010xle/AK301412) in all the cancers with one exception (Fig. 3). We found that the uc002wdm/NM_021158 (*TRIB3*) mRNA level was increased in 73.3% (22 of 30, *p* < 0.05), 50% (15 of 30, *p* < 0.05), and 93.3% (28 of 30, *p* < 0.05) cases of breast, prostate, and kidney cancers, respectively. The stable expression of uc002wdm/NM_021158 (*TRIB3*) was detected in most cases of lung cancer. The up-regulation of uc010xle/AK301412 (*ICAM1*) level was detected in breast (50%, 30 of 15, *p* < 0.05), prostate (50%, 30 of 15, *p* < 0.05), and kidney (70%, 21 of 30, *p* < 0.05) cancers. The alternative transcript uc010xle/AK301412 (*ICAM1*) was differentially expressed in lung cancer; the mRNA level of one was increased in 36.7% (11 of 30, *p* < 0.05) and decreased in 40% (12 of 30, *p* < 0.05). The expression of uc003owz/NM_001078177 *SLC29A1* transcript was not significantly changed in more than 50% cases of all tumors studied, but was up-regulated in 77.5% (31 of 40, *p* < 0.05) colorectal cancer samples (Fig. 4).

## Discussion

Alternative splicing plays a critical role in multiple cellular processes and development programs [29]. In recent years, alternative splicing has been recognized to contribute to many human disorders, including cancer [30]. Alternative splicing is one more mechanism that allows expressing transcripts involved in the regulation of apoptosis, signaling pathways and cellular metabolism [31]. Changes in splicing patterns occur widely in cancer cells and was shown to be associated with the resistance to therapeutic treatments [32]. Alterations in the trans-acting splicing regulatory elements are the most frequent in cancer [33, 34]. Mutations in various splicing regulatory factors such as U2AF1, ZRSR2, SRSF2, SF3B1, and RBM10 have been described in multiple tumor types [35–37]. Overexpression of a positive splicing factor, serine/arginine-rich (SR), was found in colorectal, thyroid, small intestine, kidney, and lung cancers [38]. On the other hand, an alternative splicing repressor, heterogeneous nuclear ribonucleoprotein (hnRNP), was primary down-regulated in these tumor types. Mutations in cis-acting splicing elements were shown in both oncogenes (*KIT*, *CDH17*, and *BRCA1/2*) and tumor suppressors (*LKB1* and *KLF6*), which have causal role in cancer initiation and progression [34, 39, 40]. Cancer-associated alterations of splicing patterns have been also reported for other cancer-related genes [39]. For instance, it has been shown that splicing pattern of Ron and Rac1 genes were altered in tumors and overexpression of their tumor-associated isoforms was sufficient to culture cell transformation [41, 42]. The alternatively spliced isoforms of MDM2/HDM2 gene, that is a regulator of p53 protein, have been identified in many cancers. Moreover, some of their protein products were shown to have transforming properties [43, 44].

Alternative splicing is known contributor to cancer pathogenesis. For instance, activation of splicing factor hnRNP by EGFRvIII mutation promotes glycolytic gene expression in glioblastoma [45]. Alternative splicing of pyruvate kinase M (PKM) pre-mRNA generates the PKM2 isoform in all cancer cells [46]. PKM2 is a critical enzyme for aerobic glycolysis that mediates Warburg effect and facilitates tumor growth [47]. PKM2 is highly expressed in embryonic and tumor cells, whereas PKM1 is primarily expressed in normal tissues [47, 48]. Splicing repressors hnRNPA1 and hnRNPA2 have been found to regulate PKM alternative splicing in cancer cells [49]. Down-regulation of these factors in cancer cells resulted in an increase in the PKM1/PKM2 protein ratio and decrease in lactate production [31]. These data indicate that alternative splicing is involved in the switch from oxidative phosphorylation to aerobic glycolysis in cancer. Mitochondrial damage modulates alternative splicing in neuronal cells leading to changes in the abundance of certain isoforms [50]. Thus, mitochondrial dysfunction, as a notable feature of cancer, may be also the mechanism underlying the changes in alternative splicing patterns.

Our study revealed tumor-associated changes in alternative splicing patterns of seven genes involved in energy metabolism, including *OGDH, COL6A3, ICAM1, PHPT1, PPP2R5D*, *SLC29A1,* and *TRIB3. OGDH* gene encodes a subunit of the multi-enzyme 2-oxoglutarate dehydrogenase complex (OGDHC) that is the first and rate-limiting component of one [51]. OGDHC plays a major role in TCA cycle and involved in the regulation of the glutamine and glutamate metabolism [52]. OGDHC is often implied to be inactive in cancer [53]. Oncogenic mutations reduce the enzyme activity of NADP+-dependent isocitrate dehydrogenases isoforms 1 and 2 (IDH1/2) resulting in increased 2-hydroxyglutarate levels and decreased concentrations of the OGDHC substrate 2-oxoglutarate [54, 55]. In a previous study, we showed that *OGDHL*, encoding one more component of the OGDHC, is down-regulated by promoter hypermethylation in CRC [56]. The promoter hypermethylation in *OGDHL* gene was also observed in breast, cervix, lung, oesophagus, and pancreas cancers [57, 58]. It has been shown that re-expression of *OGDHL* induced apoptosis through a PI3K/AKT pathway in cervical cancer cells [51]. The alterations in *OGDHC* expression were shown to be functional in various cancer cells [53]. Thus, we assumed that up-regulation of *OGDH* alternative mRNA transcript may indicate the presence of active OGDH complex in colorectal cancer that is required to control energy and glutamine metabolism.

Overexpression of *SLC29A1* alternative transcript was found in colorectal cancer and was not significantly changed in breast, lung, prostate, and kidney cancers. Transporter *SLC29A1* has been reported relating to multidrug resistance (MDR). Significant up-regulation of *SLC29A1* in colorectal, astroglial, and breast cancer cells contributed to cisplatin resistance and increased cell viability [59]. On the other hand, knockdown of *SLC29A1* reduced sensitivity of leukemia and lung cancer to drugs since it plays a role in cellular uptake [60, 61]. Increased *SLC29A1* mRNA level was suggested as a critical factor of pancreatic and biliary tract cancer cells sensitivity to chemotherapy [62, 63]. The expression of *SLC29A1* alternative transcripts in cancer has not been previously analyzed. We first observed the tumor-specific up-regulation of the uc003owz/NM_001078177 *SLC29A1* transcript in CRC. The functional role of a protein encoded by the transcript in CRC is not obvious. The further investigation will address how the transcript is associated with the resistance and sensitivity of colorectal cancer cells to therapy by various agents and may be useful for prediction of its efficacy.

Using qPCR method, we showed the tumor-specific overexpression of uc002vwo/NM_057167 *COL6A3* alternative transcript in colorectal, breast, lung, prostate, and kidney cancers. Collagen VI, a protein of the extracellular matrix (ECM), is significant in the progression of cancer and resistance to chemotherapy [64, 65]. *COL6A3* encodes one of the three α chains of type VI collagen which is involved in the regulation of metabolic health by ECM [66, 67]. Recent studies have demonstrated that *COL6A3* was up-regulated in gastric, pancreatic, and ovarian cancers [68–71]. Exon array analysis revealed the expression of *COL6A3* alternative long isoform in colon, bladder, pancreatic, and prostate cancers [72, 73].

Intercellular adhesion molecule 1 *ICAM1* (CD54) is known to play a major role in immune response, inflammation, regulation of energy balance, and angiogenesis [74–77]. Increased levels of *ICAM1* were reported in several human malignances and cancer cell lines [78]. In melanoma and gastric cancer, *ICAM1* expression was associated with an increase in metastases [79, 80]. This can be explained by ICAM1-mediated activation of leukocytes and induction of cell migration [81]. On the other hand, immunohistochemistry studies reported better prognosis for patients with ICAM1-positive tumors (including lymphoma, ovarian, colorectal, head and neck cancers) [82–84]. Cancer cell can expresses and release soluble ICAM1, that is regulated by TNF-α and INF-γ [85]. It is an essential mechanism used by tumors to escape immune recognition [86, 87]. For example, elevated serum levels of ICAM1 in colorectal cancer patients were correlated with tumor stage and tendency to metastasis formation [84, 88, 89]. Thus, ICAM1 seems to have different roles in tumorigenesis. Tumor-specific expression of *ICAM1* alternative splice variants was not previously reported. We found up-regulation of uc010xle/AK301412 *ICAM1* alternative splice isoform in colorectal, breast, prostate, and kidney cancers that may be important prognostic factor.

*PHPT1* and *PPP2R5D* genes encode proteins belonging to phosphatase activity and glucose metabolism [90–92]. *PHPT1* has been found to be overexpressed in lung cancer and playing a role in cancer progression, migration and invasion [93, 94]. *PPP2R5D* gene, encoding a regulatory B subunit of protein phosphatase 2A (PP2A), was reported to be involved in Myc activation and degradation [95]. We first showed up-regulation of *PHPT1* and *PPP2R5D* alternative transcripts in colorectal cancer.

The protein encoded by *TRIB3* gene is tribbles pseudokinase-3 that has been proposed as inhibitor of AKT and interaction partner of transcription factors (including ATF-4, CHOP9, and several MAPKs) that regulate cell growth, differentiation and metabolism [96–100]. Schwarzer and co-authors showed that TRIB3 emerges as a transcriptional target of PI3K/Akt signaling pathway and is involved in regulation of glucose metabolism [101]. Recent studies reported that increase in *TRIB3* expression promoted cancer cell death through apoptosis [102–105]. Genetic inhibition of *TRIB3* resulted in activation of mTORC2/AKT/FOXO pathway and was associated with more aggressive phenotype in several animal models of cancer [106]. However, *TRIB3* was up-regulated in CRC samples, gastrointestinal and colorectal cancer cell lines [107]. These data are consistent with the overexpression of both *TRIB3* alternative splice variants in CRC observed in this study. Thus, tumor-associated changes in alternative splicing lead to overexpression of certain *TRIB3* splice isoforms which can be involved in development of colorectal cancer.

## Conclusion

In the present study, using our previously developed bioinformatics tools and TCGA data, we evaluated alternative splicing profiles of genes associated with energy metabolism in CRC samples and then validated the results by qPCR. Differential expression of the transcripts of seven genes (*OGDH, COL6A3, ICAM1, PHPT1, PPP2R5D, SLC29A1,* and *TRIB3*) was confirmed. Alternative transcript uc003owz/ NM_001078177 of *SLC29A1* was characterized with tumor-specific overexpression in CRC that can be associated with drug resistance and sensitivity. Changes in alternative splicing patterns of *OGDH* gene may play an important role in the regulation of energy and glutamine metabolism in CRC. Overexpression of *COL6A3* alternative transcript in all examined tumor types indicates its significant contribution to disease development and pathogenesis. Increase in expression of *PHPT1*, *PPP2R5D*, and two *TRIB3* transcripts indicates that tumor-associated changes in alternative splicing can affect glucose metabolism in colorectal cancer. Several alternative transcripts may be suggested as potential cancer biomarkers, although further studies must be performed to confirm these results.

## Additional file

Additional file 1: Table S1. Differential expression profiles of genes and transcripts in colon adenocarcinomas compared to matched normal tissues (TCGA data analyzed by CrossHub). (XLSX 43 MB)
